# An Urban Intelligence Architecture for Heterogeneous Data and Application Integration, Deployment and Orchestration

**DOI:** 10.3390/s24072376

**Published:** 2024-04-08

**Authors:** Stefano Silvestri, Giuseppe Tricomi, Salvatore Rosario Bassolillo, Riccardo De Benedictis, Mario Ciampi

**Affiliations:** 1 Institute for High Performance Computing and Networking, National Research Council of Italy (ICAR-CNR), Via Pietro Castellino 111, 80131 Naples, Italy; salvatore.bassolillo@icar.cnr.it (S.R.B.); mario.ciampi@icar.cnr.it (M.C.); 2 Department of Engineering, Università degli Studi di Messina, Contrada di Dio 1, 98166 Messina, Italy; 3 CINI—Consorzio Interuniversitario Nazionale per l’Informatica, Via Ariosto 25, 00185 Roma, Italy; 4 Department of Science and Technology, University of Naples Parthenope, Centro Direzionale, Isola C4, 80143 Naples, Italy; 5 Institute of Cognitive Sciences and Technologies, National Research Council of Italy (ISTC-CNR), via Giandomenico Romagnosi 18/A, 00196 Roma, Italy; riccardo.debenedictis@istc.cnr.it

**Keywords:** urban intelligence, smart cities architecture, data platform, data integration, smart cities workflows, digital twins deployment, digital twins integration

## Abstract

This paper describes a novel architecture that aims to create a template for the implementation of an IT platform, supporting the deployment and integration of the different digital twin subsystems that compose a complex urban intelligence system. In more detail, the proposed Smart City IT architecture has the following main purposes: (i) facilitating the deployment of the subsystems in a cloud environment; (ii) effectively storing, integrating, managing, and sharing the huge amount of heterogeneous data acquired and produced by each subsystem, using a data lake; (iii) supporting data exchange and sharing; (iv) managing and executing workflows, to automatically coordinate and run processes; and (v) to provide and visualize the required information. A prototype of the proposed IT solution was implemented leveraging open-source frameworks and technologies, to test its functionalities and performance. The results of the tests performed in real-world settings confirmed that the proposed architecture could efficiently and easily support the deployment and integration of heterogeneous subsystems, allowing them to share and integrate their data and to select, extract, and visualize the information required by a user, as well as promoting the integration with other external systems, and defining and executing workflows to orchestrate the various subsystems involved in complex analyses and processes.

## 1. Introduction

The concept of a Smart City is associated with an urban context, where the integration and cooperation of cyber-physical systems (CPS) with the workflows for managing city life and its citizens enable the analysis and automated management of aspects related to them. The Smart City has emerged as a beacon of innovation, efficiency, and sustainability, representing a functional and structural improvement, where advanced technologies and data-driven approaches are harnessed to enhance quality of life, safety of citizens, and the sustainability and efficiency of urban centers [1,2]. This urban model integrates cutting-edge technologies, such as Internet of Things (IoT) devices, big data analytics, artificial intelligence, and connectivity to optimize infrastructures, transportation, energy, healthcare, governance, and other services [3,4].

Among these technologies, digital twins (DTs) [5] have recently become an essential tool for the proper management of modern cities, providing real-time monitoring and more effective decision-making processes [6,7,8]. A DT is a virtual representation of a physical entity or system, faithfully mirroring its behavior, characteristics, and interactions in real-time, thanks to the integration of sensor data, computational modeling, and advanced analytics [9]. DTs offer a powerful paradigm for modeling, simulating, and understanding the complex systems [9,10] that run alongside real-time processes in different fields of application, including healthcare, environmental monitoring, aerospace engineering, robotics, smart manufacturing, renewable energy, and process industry [11,12,13,14].

At the forefront of this urban transformation provided by Smart Cities lies the integration of cutting-edge technologies into the urban fabric, giving rise to the urban intelligence (UI) paradigm, which proposes an ecosystem of technologies to improve the urban environment, well-being, quality of life, and smart city systems [15]. These UI systems represent a paradigm shift in urban management, leveraging DTs, as well as data-driven insights, artificial intelligence, and interconnected networks to enhance the quality of life for citizens, while optimizing resource utilization. The adoption of UI based on DTs, and the integration of CPS, IoT, and data of the city, provide a cyber-physical counterpart to city systems, to increase the sustainable growth of the city and strengthening its functions, while ensuring the enhanced quality of life and health of the citizens [16,17]. Specific structures and IT technologies are necessary for the proper functioning of the DT context: network infrastructure, dedicated IoT sensor networks, information systems, and high-level web services, such as cloud or edge-computing environments [18], are required to collect and manage the heterogeneous data of the city [19,20] and to provide a proper hardware and software infrastructure to allow the execution of the required models, visualizations, and user interface functionalities [21,22].

Obviously, the complexity and heterogeneity of the collected data and the involved technologies of UI subsystems require an advanced IT platform that is able to effectively deploy and integrate the various systems belonging to the CPS, providing at the same time proper data management and sharing capabilities and effectively supporting information searching and retrieval, offering visualization and user interface capabilities, and fully and effectively integrating DTs [15,23]. Middleware and frameworks dedicated to Smart Cities simplify the deployment, development, and management of the applications and subsystems of the UI, but their definition is not easy. Therefore, specific solutions must be developed, easing the integration of heterogeneous subsystems, their deployment, and supporting at the same time the collection, management, and sharing of multiple data sources and formats. Moreover, orchestration and coordination functionalities are required to manage the data and process flows involved in complex analyses, when several subsystems must interact to obtain the requested results [24].

This paper presents the architecture of an IT platform to address the aforementioned issues. The architecture is able to support the design, integration, deployment, and management of DTs and other subsystems of a UI. It also provides functionalities for the acquisition, sharing, and integration of data, as well as for the visualization of analyses and the realization of dashboards. Finally, it includes a workflow engine to design and execute the workflows required for orchestrating and coordinating the subsystems, and processes and data flows for each specific analysis and simulation where the interaction of different subsystems is involved.

The proposed architecture provides resources for the deployment and orchestration of container-based implementations of DTs and other subsystems of a complex UI environment. Moreover, it includes a layer specifically tailored to provide data storage, management, integration, and retrieval functionalities. This layer also acts as a data collector, facilitating the sharing of data and subsystem integration. The proposed architecture also provides a mechanism to design, manage, and orchestrate the interactions and data flows of these components, exploiting a specific workflow engine layer. The upper layer offers data visualization capabilities, providing the resources to realize dashboards to interact with the UI system and show the results of analyses of aspects of the city. Finally, a transversal communication interface layer is available, based on an application program interface (API), that allows data sharing among the subsystems of the UI and with external systems. This layer also provides authentication and authorization mechanisms, respecting the requirements of the Italian Public Administration IT systems interoperability to facilitate interconnection of the UI with other public IT systems.

The implementation of the proposed architecture is based on open-source technologies and was successfully adopted and tested in a real-world UI system in Italy, demonstrating its usability and effectiveness.

In summary, the main contributions of this paper are as follows:

- an architecture for the deployment and integration of the subsystems of an UI model, which can be implemented using open source technologies and frameworks;
- the availability of a data platform that acts as a common data collector, facilitating data integration and sharing within the UI internal modules and with external IT systems, whose API also meets Italian requirements for public administration IT system interoperability, following the principles articulated in the New European Interoperability Framework (EIF) [25];
- the integration of a workflow engine that is capable of designing, coordinating, and orchestrating data and process flows between the UI subsystems, when complex interactions are required to obtain specific information and/or functionalities.

The remainder of this paper is structured as follows: Section 2 presents an analysis of the literature concerning the main concepts concerning the considered scenario. Section 3 provides a high-level overview of the proposed architecture, while Section 4 delves into the main mechanisms and the common workflows of the solution. Section 5 describes the tools and the frameworks used to implement the architecture. Section 6 presents a use-case scenario, where the proposed architecture was exploited for both data and system integration in a real-world setting. Finally, Section 7 summarizes the conclusions, highlighting the contributions of the paper and detailing future works.

## 2. Related Works

In the era of Smart Cities, where urban landscapes are becoming increasingly interconnected and data-centric, research encounters numerous complexities, notably in managing the diverse nature of data [26], as well as facilitating the design, integration, deployment, and management of heterogeneous systems [24,27]. The cornerstone of an urban intelligence platform is the integration of data collection, analysis, and decision-making processes, as underscored in [28,29,30,31]. The deployment of urban intelligence platforms within the ambit of smart cities, however, is not devoid of challenges and limitations. Foremost among these is the imperative to navigate critical data and technology management issues to unlock the full potential of urban informatics, ensuring the agility, adaptability, and scalability of the city [30,31].

Focusing on data management solutions for Smart Cities, data lakes have emerged as a pivotal infrastructure, shaping the foundation for the effective utilization of vast and heterogeneous datasets generated by a Smart City initiative. Recent strategies employed in data lakes often integrated both SQL and NoSQL database approaches, incorporating online analytics processing (OLAP) and online transaction processing (OLTP) capabilities [32]. In contrast to the hierarchical structure of data warehouses, with their file or folder-based storage, data lakes adopt a flat architecture. Here, each data element is assigned a unique identifier and a set of extended metadata tags, accommodating data of various shapes and sizes and emphasizing the preservation of the order of data arrival. Conceptually, a data lake can be envisioned as a vast reservoir, amalgamating historical data and new data—whether structured, unstructured, semi-structured, or binary from diverse sources like sensors and devices—into a unified repository. Notably, the schema and data requirements are not predefined; they materialize only when the data are queried, providing a dynamic and flexible approach to data management. In [33], the authors studied the features and capabilities of NoSQL technologies when applied to the implementation of a data lake for Smart Cities, considering performance, scalability, accuracy, and complexity as the main indicators. The results of their experiments revealed that MongoDB was the most stable NoSQL database when assessing its use in the implementation of a Smart City data platform.

The data lakes used in a Smart City frequently incorporate a semantic database, a conceptual model that utilizes standardized technologies akin to those employed in constructing Internet hyperlinks. This model adds a contextual layer to the data, elucidating the meaning of the data and establishing their interconnections with other datasets. An example of a semantic data lake tailored for a Smart City was described in [34], where the authors included a semantic metadata catalog on top of the data lake, leveraging tools and metrics to ease the annotation of the data lake metadata, as well as modeling indicators and analysis dimensions and exploiting a multi-dimensional ontology to check their conformance. Finally, they applied an enrichment of the indicators based on a customization of the profiles and preferences of the users, further facilitating the exploration of data. Experiments on the Smart City domain confirmed the effectiveness and the feasibility of this approach. The authors of [35] presented a data marketplace based on semantic data management, which allows different stakeholders (public institutions, enterprises, citizens) to easily share the data of a smart city. In detail, data providers must annotate their data following specific semantic models, which helps the data sources to be found and understood by data consumers, promoting integration and use in their application. In [36], creating a semantic data layer over Smart City data sources was proposed. This approach was based on a procedure to build and manage a semantic layer and included a semi-automatic tool to facilitate the construction of this semantic layer and the annotation of data. The proposed approach was successfully applied in the lexical enrichment of the data source attributes of a smart city. A platform for environmental Smart City data was presented in [37], which was able to share, search, store, and visualize heterogeneous data, easing their accessibility and enriching them with metadata and geographical information. Moreover, elastic stack technologies were integrated to improve the real-time capabilities of the proposed platform.

Real-time analytic capabilities are often required in the case of data lakes and other frameworks and for the middleware that empowers Smart Cities to respond promptly to dynamic urban challenges. Whether it is traffic management, energy consumption optimization, or emergency response coordination, the agility afforded by data lakes ensures that decision-makers are equipped with timely and actionable intelligence [38]. Solutions to increase the real-time performance of data lakes for smart cities were recently described in [39,40,41], adopting and integrating big data technologies and frameworks, such as Hadoop, Spark, Solr, HDFS, and Hue, while defining architectures specifically tailored for Smart City requirements and features.

As mentioned above, UI platforms must also harness the potential of emerging technologies, such as the Internet of Things (IoT), DTs, AI, and other systems, to assimilate and scrutinize data across a city’s myriad systems and subsystems [30,42]. This can be achieved through the efficient orchestration of services and infrastructures, as well as fostering enriched interactions between businesses and citizens [42,43]. Therefore, the definition of specific architectures and middleware able to facilitate and support not only data management and integration but also the deployment and orchestration of the other subsystems of the UI, including the data lake itself, is a challenge faced in recent literature using various approaches. In [24], a review of the most recent Smart city middleware was presented, analyzing their enabling technologies, non-functional requirements, architectural styles, and programming paradigms, and highlighting challenges and open issues. Moreover, the authors framed a conceptual framework for smart city middleware whose purpose is to define its essential features and facilitate the creation of integrated and scalable smart city applications.

The authors of [44] described a platform capable of both promoting the integration of heterogeneous data and supporting the development of smart city applications. In detail, this platform integrates urban data with geographical information and relies on a dedicated middleware infrastructure, whose architecture includes a layer for context data management, a layer for the integration of heterogeneous data (which also includes a semantic support and an IoT manager), a layer for data analysis and visualization, and finally resources for data security and privacy. The implementation of this middleware leverages several different technologies, mainly based on Java and WildFly for the core functionalities; Vue.js for the dashboards; Spring for the interconnection with third-party systems; and Flink, HDFS, Kafka, Jean, PostGIS, and MongoDB for the data management, integration, and analysis functionalities. The experiments performed in a real smart city setting allowed the authors to confirm the performance and scalability of this platform. In [45], a large-scale data analytics framework for Smart Cities was described, which is capable of supporting the creation of smart city services and providing a distributed system for semantic discovery, data analytics functionalities, and interpretation of large-scale real-time IoT data. Its main features include a unified view of the data, the integration of data analytics modules, and the ability to perform data aggregation, event detection, quality assessment, contextual filtering, and decision support. Implementation in a use-case scenario demonstrated the effectiveness of this framework. An open-source platform that provides APIs and tools for the development, among others, of Smart Cities applications was described in [46]. In this case, the authors proposed an architecture that manages IoT resources and data ingestion at the lower layers and then integrates these data, while also providing semantic enrichment. Finally, the data analytics and security and privacy layers include these kinds of services.

Issues related to the management of the numerous and multiple sources of information and embedded systems involved in a Smart City, and how to share data and merge the subsystems into a single infrastructure, were addressed by the computational platform described in [47]. In detail, the study analyzed the capabilities of an autonomic reflective middleware for these purposes. This middleware is based on intelligent agents and uses ontologies for the adaptation to the specific context and to respond to unforeseen situations. The authors of [48] presented a platform for the integration of the diverse and heterogeneous data sources of a Smart City, capable of also incorporating geographical data and supporting a semantically enriched data model by means of ontology classes and properties for effective data analysis and integration. Moreover, the platform facilitates the deployment of Smart Cities applications, offering a suite of high-level services, which can be repurposed by developers.

The main innovations of the architecture proposed in this paper with respect to the recent literature are related, first, to the inclusion of a layer to support the deployment and orchestration of containers that implement the various subsystems (DTs, CPS, data platform, etc.) of a UI model; moreover, it also provides a workflow engine to allow the design and orchestration of their interaction and data flow. In this way, it not only facilitates data integration, exploiting a data lake approach, but also the integration of the modules in a single framework. Finally, it includes a communication interface that follows the Italian guidelines for interoperability with public administrations and third-party IT systems (following the principles of the New European Interoperability Framework), guaranteeing the required level of security.

## 3. Architecture

**Figure 1:**
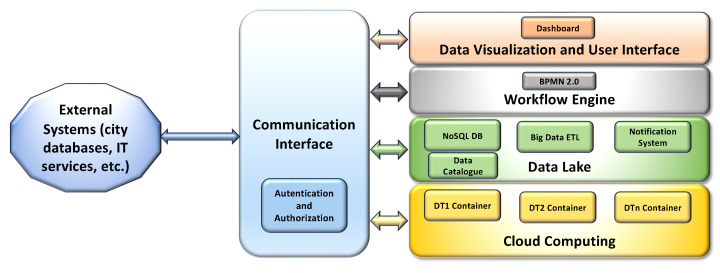
Layers of the proposed IT template architecture.

The layers of the proposed IT platform architecture are depicted in Figure 1.

The cloud computing layer is the lowest layer, which provides and dynamically manages and coordinates the hardware and software resources in the cloud environment. This layer is also devoted to the deployment and orchestration of containers, facilitating the installation and execution of the containerized software modules that implement DTs and the other subsystems in a cloud-based environment, as well as facilitating their integration with other systems.

The next layer is the data lake, whose purpose is to acquire, store, integrate, query, and retrieve data collected or produced by any UI subsystem, as well as obtained from external sources. The data lake is based on a NoSQL database, which also integrates a distributed file system and basic big data analytics (BDA) functionalities. This layer is able to manage and store large volumes of structured, semi-structured, and unstructured raw data, even if produced at high rates. To prevent data swamp degeneration and support the integration of heterogeneous data, a data catalog; metadata enrichment; and BDA extract, transform, and load (ETL) functionalities (based on the approach described by [49]) are also included in the data lake layer, with the purpose of enriching the heterogeneous data with specific metadata, extracting and integrating the required information, and providing a catalog that can be queried, to facilitate the integration of the internal modules and with external IT systems.

More in detail, the data are stored in the Data Lake enriched, when required, with additional metadata, which not only include general purpose information such as the time and date but also more specific details, such as the range of possible values of data, the unit of measurement, the source, and the type. Moreover, an additional collection of the NoSQL DB is used as a data catalog, where the details of each kind of acquired data are stored in a document, providing a brief description of the data, a list of the fields with a corresponding description, and the DB and collection of the NoSQL DBMS where it is stored. The data catalog can be queried to obtain the details of the data structures and their semantics, facilitating data exchange between the internal modules of the UI, as well as integration with external systems, and finally easing the customization of ETL functionalities, in the case of new data that must be included in the UI.

The data lake layer also offers basic ETL functionalities specifically tailored for data aggregation and integration. Exploiting a BDA engine, which is capable of supporting high data processing rates, the information included in the data is extracted, transformed, aggregated, and stored in new collections, following the requirements of the various DT modules of the UI, optimizing the processing time and supporting the information exchange. In this way, the data lake layer also acts as a common data collector for the DTs and other subsystems, allowing them to easily share their data and communicate with each other.

Finally, asynchronous notification services are included in this layer, for notifying specific software modules (or users) of possible data updates in the data lake, according to a publish/subscribe pattern, thus optimizing the bandwidth and performance of the DB.

The workflow engine layer includes a workflow management and execution component aimed at providing the UI system with an automatic business process engine, whose purpose is to easily implement and execute the workflows that are used to model each analysis requested, where the interaction of more subsystems is necessary. The workflows are represented using the Business Process Model and Notation (BPMN) OMG standard [50] for business process modeling. This standard provides a graphical notation for specifying business processes in a business process diagram, based on flowcharting, with the main purpose of supporting business process management. The semantic enhancements found in BPMN 2.0 allow it not only to model the business processes but also to model and execute the integration processes between different software systems, coordinating and orchestrating them by executing the workflows and exploiting dedicated workflow engines [51]. Therefore, this layer also includes a workflow engine, which is used to coordinate the process and data flows through the various subsystems to obtain the required information or to perform the requested operation when different subsystems are involved in the process.

The upper data visualization and user interface layer provides the visualization of the integrated data and user interface features (including dashboards), adopting a web-based approach.

Finally, communication between the data lake and the other UI subsystems exploits a vertical communication interface layer, where a set of dedicated APIs are available to provide a common and standardized way to obtain or send data and access the exposed functionalities. The communication interface layer also offers the possibility of connecting the IT Smart City template with external systems of the city (like city databases or other IT services). Finally, it includes the required authentication and authorization functionalities, which are able to implement single-sign-on through standardized claims.

In addition, the proposed architecture includes some standard workflow templates, which aim to formalize the more common coordination actions that are required in most cases of UI implementation.

## 4. Workflows

**Figure 2:**
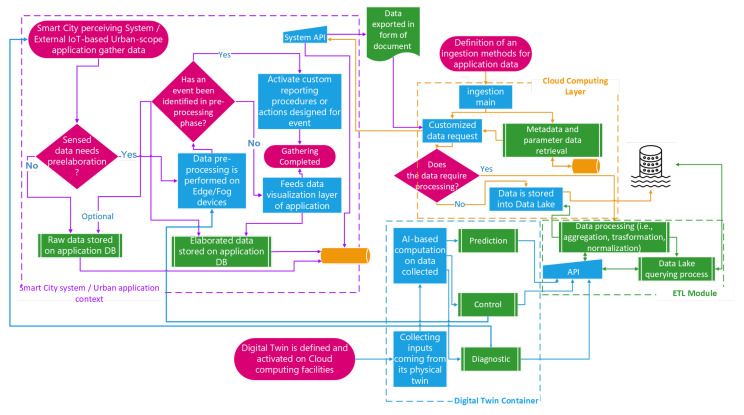
Workflow representing the data lake’s ingestion of data.

**Figure 3:**
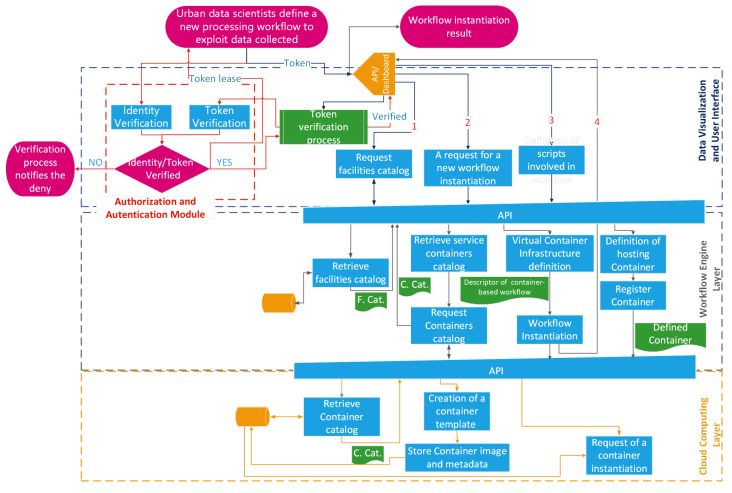
Instantiation of a new application-level workflow operated by an urban data scientist.

**Figure 4:**
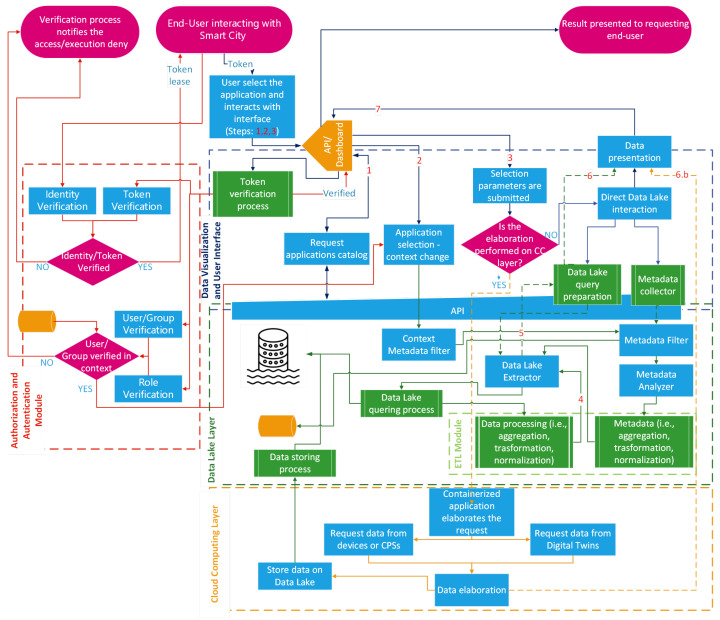
Interactionof a user with the data visualization layer.

On the way to developing a practical template for the realization of an urban intelligence system, we introduce some workflows as templates to design the most useful operational activities. The workflows described in the following cover the three main activities involving a Smart City:

- *Data ingestion from Smart City and urban applications*: the flows of data moving towards the data lake are generated on the basis of specific ingestion workflows, which are responsible for gathering data from their sources (e.g., UI subsystems, external systems, and so on). To achieve this goal, the cloud computing layer uses its container management platform to execute the software modules that perform the workflow of gathering data from a specific source. The workflow in Figure 2 shows how an urban data scientist might interact with the template system to create the above-discussed data flow.
- *Instantiation of a new application-level workflow operated by an urban data scientist*: the cloud computing layer is involved in the execution of containers devoted to performing the extraction, aggregation, monitoring, and processing of data stored in the data lake. An urban data scientist interacts with the workflow engine through the data visualization and user interface layer to design an appropriate workflow. The aim is to correlate the data stored in the data lake, process them, and generate opportune actions as a consequence of the conditions obtained as input. This workflow is depicted in Figure 3.
- *End-user interaction with a Smart City platform through the data visualization layer*: Data have to be requested by the end-user (e.g., a citizen) through the “Data Visualization and User Interface” layer. To achieve the goal of offering a reporting system for citizens who are interested in certain Smart City characteristics (i.e., citizens interested in the identification of a shaded route that allows them to visit specific points of interest), the workflow representing the facility offered to a Smart City user is depicted in Figure 4.

### 4.1. Data Ingestion from Smart City and Urban Applications

The data lake needs to be continuously fed with data flowing from the Smart City, considered as a set of CPSs and DTs, constructed and defined by Smart City scientists, to monitor one or more urban scenario or device. Figure 2 shows the workflow describing the main tasks producing data that feed the data lake layer, which is driven by components running into containers hosted by the cloud computing layer. As depicted, the data sources mainly fall into physical and virtual systems.

The former represent an urban CPS or application, exploiting the Smart City infrastructure’s features to perceive data representing bare or elaborated environmental characteristics. In this case, a data scientist enables a Smart City system to ingest data by defining and running one or more containers through the cloud computing layer to orchestrate a time-based data-gathering process.

The latter represents the containerized components intended to act as a DT of controlled devices, IoTs, or a whole CPS operating in the Smart City as an isolated device or as part of a complex environment. The workflow in Figure 2 shows a partial view of the internal data management performed by an urban-scale application or a Smart City system.

The data perceived are pre-processed and stored in a repository (represented by the process “*Raw data stored on application DB*”); if they are pre-processed, the identification of specific events enables appropriate actions, countermeasures may be put in place, or otherwise the events may be reported (represented by the step named “*Activate custom reporting procedures or actions designed for events*”).

Obviously, the duties connected to urban-scale applications or Smart City systems are not only the ones reported in the figure, but they represent precisely what is required for the data ingestion, which is driven by the logic put in place by data scientists in the form of containerized application in the cloud computing layer. Here, a process to gather data from Smart City sources is run (it is mostly time-based) and is meant to enrich those data with metadata. Moreover, if a supplementary data elaboration step is needed, the logic sends the data gathered to the ETL module of the data lake layer to realize the appropriate operations. This section of the flow is illustrated within the orange dashed rectangle, symbolizing the workflow related to the ingestion method specified for application data. These data are predominantly isolated within the cloud computing layer elements but interact with other segments of the architecture solely for specific data requests directed to the Smart City system and for setting or storing the output of data processing in the data lake.

The cases of containerized DTs are different. By nature, a DT receives data representing inputs, outputs, and internal states of its physical twin (a CPS or simply the physical object under analysis) to perform all or a part of the following three kinds of duties: prediction, control, or diagnostics. The outputs of DTs contain valuable data for the Smart City system. Therefore, it is necessary to store these data in the data lake after undergoing an elaboration phase defined within the ETL module. Additionally, apart from these interactions, DTs generate control outputs that impact the data preprocessing block within the *urban application context*. Furthermore, DTs may produce diagnostic output mechanisms capable of interacting with the Smart City.

### 4.2. Instantiation of a New Application-Level Workflow Operated by an Urban Data Scientist

The urban scenario, dynamic in nature, constantly evolves, both from an infrastructure point of view (e.g., edge devices), which may be subjected to update, extension, or substitution, and from an application point of view, which is enriched by a plethora of applications satisfying citizen requests. For this reason, an urban data scientist needs to adapt/normalize/elaborate data according to the application level requirements; a solution is offered via a dashboard-mediated interaction with the workflow engine layer, to define an application-level workflow. Due to the interaction of the dashboard, the platform has to lease a token to the data scientist as a result of the preliminary identity verification process, in Figure 3, a red dashed rectangle surrounds this part and red arrows depict the involved interactions. This token, till its expiration, is used by the platform to grant access to the requested resources.

The data scientist defines the application-level workflow after three main interactions with the platform:

1. verification of facilities already available to the data scientist and all services available in the container catalog; in Figure 3, this interaction is represented by the flow starting with arrow 1;
2. definition of a workflow producing a new facility as a composition of pre-existent facilities, pre-existent services, and/or new custom services; in Figure 3, this interaction is represented by the flows starting with arrows 2 and 4;
3. (optional) in case of a definition of a new custom service, the third step is used to define new functionalities in the form of scripts; in Figure 3, this interaction is represented by the flow starting with arrow 3.

The former step is meant to identify whole facilities or (*atomic*)-services available for the creation of a new workflow; the data scientist interacts with the graphic user interface (GUI) offered by the dashboard to inspect the elements to be included inside the workflow. It is clear that the GUI has to collect all the information related to the catalog, offering useful elements (i.e., facilities from the workflow engine layer, service containers from the cloud computing layer) that, if selected by the data scientist, will become part of a new workflow. The retrieval processes of these elements are performed by the workflow engine layer as the reaction of some API-based requests coming from both *“Request facilities Catalog”* and *“A request for a new workflow instantiation”* interaction blocks, as shown in Figure 3.

Moreover, a data scientist may tailor ad hoc services to generate (or complete) the design of a new facility, and this is possible with a two-step procedure that corresponds to the latter of the previous enumerations, the third (optional-) interaction; this sub-flow is described by the activities performed by the “*Workflow Engine layer*’s” block named “*Definition of hosting Container*”. The tailoring activity is meant to define the minimal characteristics of the hosting container (e.g., resources, networks, volumes, environmental variables, capabilities, and so on) and the activities performed during the container life cycle: this is realized in the form of scripts injected into the container volume that will be executed as an activation command of the container itself.

Finally, when the data scientist has all the elements needed to define the application-level workflow, he can exploit the second interaction. As a consequence of this action, the platform, through the workflow engine, composes the descriptor of the workflow (i.e., in the Figure 3 is named “Descriptor of container-based workflow”). The descriptor thus generated is the input of the “Workflow Instantiation” process, which is enrolled in container instantiation on the cloud computing layer via API requests. The data scientist is notified about the workflow instantiation result (in Figure 3 this is represented by the arrow with label 4); in this way, the data scientist receives helpful information for further identification/interaction with the realized workflow.

### 4.3. End-User Interaction with Smart City Platform through the Data Visualization Layer

A citizen or, in general, an end user may interact with the Smart City platform to exploit the available applications. As a first step, the user has to be recognized by the “Authorization and Authentication Module”; if the identification process is successfully completed, a token granting access to the platform is provided; in Figure 4, a red dashed rectangle surrounds this part, and red arrows depict the involved interactions. The user exploits the application of the platform via a three-step procedure:

1. the user chooses an application from the “Application Catalog”, in Figure 4 this interaction is represented by the flow labeled with arrow 1;
2. the platform operates a context switch towards the selected application, in Figure 4 this interaction is represented by the flow labeled with arrow 2;
3. the user interacts with the application by submitting the requested parameters, in Figure 4 this interaction is represented by the flow labeled with arrow 3.

From the operational point of view, the first two steps are tightly coupled. Indeed, the selection made in step 1 enables the exploitation of an application by the user that, even if authenticated, it may not be possible ti interact with (i.e., the user belongs to a group that is not authorized). As a result of an affirmative permission check, an interaction with the data lake layer, involving both the “Context Metadata filter” and the “Metadata filter”, produces a context switch on the data visualization layer that enables the user to select the application input parameters.

The third step produces a different behavior according to the application. In the proposed platform, the applications offered to the users exploit two kinds of services, as it is possible to deduce from Section 4.1 and in Section 4.2, that are supported by functionalities relying on different layers:

- on the data lake, in the form of pre-defined data lake interrogations acting from the conceptual point of view akin to a prepared statement, in Figure 4 this interaction is originated by the flow tagged with arrow 4;
- on cloud computing, in the form of the service invocation offered by the containerized workflows (predefined or custom) instantiated according to the procedure described in Section 4.2.

In this way, in the case of an application relying on data lake facilities, the answer for the user (shown in Figure 4 by the arrow 7) is generated downstream through the involvement of the data lake query preparation process, which collects all the search keys, metadata, and aggregation/transformation/normalization constraints needed for retrieving and presenting the data obtained with the flow tagged with label 5 to the requesters. This data require that an appropriate adaptation is made by the *data lake query preparation* in the *data visualization and user interface layer*, which is represented by the arrow labeled with 6.

In the second case, the workflow is a little different because it mostly exploits a long-term data-requesting interaction:a user requests a similar functionality if he is setting up a service that will produce exploitable information and data over time. This procedure causes the instantiation of one or more containers on the cloud computing layer, to set up the target workflow, which offers the requested facilities. The outcomes of the workflow are presented to the requesters (see the flow tagged with label 6.b shown in Figure 4) and, at the same time, stored in the data lake for future purposes.

Finally, new customized workflows, based on the specific requirements of the UI, can be easily defined, implemented, and executed by exploiting the functionalities offered by the workflow engine layer of the proposed architecture.

## 5. Implementation

This section presents the technologies adopted to implement the various layers of the proposed architecture. The cloud computing layer relies on Kubernetes [52] to orchestrate and deploy the containers, which are based on Docker technology [53].

The data lake layer includes three main components: a NoSQL database, a notification system, and a data catalog. The NoSQL database, which is able to store, manage, and process structured, semi-structured, and unstructured data, is based on MongoDB [54]. This NoSQL DB guarantees the required level of scalability and performance for Smart City applications [33]; a scheme-free data structure; the required ETL, integration, retrieval, indexing, and querying functionalities; and can also manage geojson data, facilitating the integration of geographical information of the city into the data platform of the architecture [55]. Moreover, a distributed file system (DFS) is implemented in this layer, allowing the storage of huge volumes of large raw binary data (images, etc.). In this case, GridFS [56] is adopted, being natively integrated with MongoDB and able to exploit its indexing and scalability functionalities [57].

To prevent data swamp degeneration of the data lake, and to support the integration of heterogeneous data, the collected data are enriched with specific metadata. The same process is also performed in the case of the raw binary files stored in the DFS, including in the DB a corresponding entry and metadata for each file. The metadata enrichment process is directly implemented in the API in the communication interface layer, and the lists of the metadata of each kind of data are included in the data catalog.

Due to the high heterogeneity of the data stored, the data lake also offers basic ETL functionalities for data aggregation and integration, with the purpose of supporting the data exchange between the various subsystems of the smart city. The simplest ETL functionalities are directly included into the data lake API. If higher performances are required for ETL and data aggregation, we also include a big data analytics framework to speed up the most complex requests to the database [58], and ETL functions [49] are also available. For this purpose, we adopt Apache Spark and SparkSQL [59], which is integrated within MongoDB using the Spark MongoDB connector and a specifically developed Scala code. Its purpose is to implement a set of big data ETL and querying functionalities on large data collections stored in MongoDB, following the method previously described in [49] and tailored for the proposed architecture, which allows further improving MongoDB performance in the cases of very large data collections and complex aggregations.

A data catalog is also included in this layer, with the purpose of sharing the details and the semantics of the data stored and integrated, facilitating in this way the retrieval of data and the integration of the platform with other systems. Moreover, the data catalog also lists the metadata. Its implementation uses a collection of the NoSQL DB, which contains a document for each type of data and metadata set stored.

Finally, the data lake layer also offers asynchronous notification of data updates, adopting a publish/subscribe approach, provided by the Message Queue Telemetry Transport (MQTT) standard protocol [60], using the Eclipse Paho MQTT Python library [61] version 1.6.1. In this way, it is possible to select the data that require notification and the corresponding subscribers (software modules or users) that receive the notifications. In the proposed architecture, the data lake also has the purpose of acting as a data collector for all kinds of data of the urban intelligence. In this way, it allows any module (DTs, etc.) to easily share their own data and exchange them with the other subsystems. The asynchronous notification services included in the platform can improve the data exchange process, notifying specific modules of possible data updates, according to the publish/subscribe pattern, and optimizing in this way the bandwidth and the performances of the DB.

The workflow engine layer leverages Flowable [62], a workflow management and engine platform based on BPMN 2.0 standards, which allows workflows to be designed and published, as well as triggered manually, in scheduled mode, or hooked into raised platform events.

The data visualization and user interface are based on Vue.js [63], a progressive JavaScript framework for building user interfaces, with a focus on simplicity and flexibility, and featuring a reactive data-binding system and a component-based architecture, and Vuetify [64], a material design component framework for Vue.js, providing a set of predesigned and customizable UI components that follow material design guidelines.

Finally, the communication layer allows communication between the data lake and the other subsystems of the UI, as well as with external systems. This vertical layer exploits a REST interface, where a set of dedicated and standardized APIs are available to provide a common and standard way to obtain or send data to the data lake and to access and execute the exposed functionalities. This REST API is implemented using the Flask Python library [65]. The interface provided by this layer, in conjunction with the workflow engine, implements the interconnection between the modules and subsystems of the smart city, exploiting in this way the proposed platform to facilitate their integration into a framework. Moreover, the interface also offers the possibility to connect the platform with external systems (such as databases or external IT services and systems). In this case, the API respects the requirements of the Italian ModIPA standard (*Modello di Interoperabilità per la Pubblica Amministrazione*—Model for the Interoperability of Public Administrations) [66], which gives a set of guidelines, technologies, and standards to promote interoperability among public administrations’ IT systems and between them and third parties, by means of technological solutions that ensure interaction and exchange of information without constraints on implementations, respecting privacy and security requirements, and following the principles articulated in the New European Interoperability Framework (EIF) [25,67,68]. In detail, this guideline proposes the adoption of REST API for the data exchange among public administrations and third parties, including security, authentication, and authorization mechanisms, and listing a set of possible technical solutions. In our case, the security of access and authorizations are guaranteed by the use of OAUTH 2.0 protocol [69], implemented using Keycloak [70], an open-source identity and access management framework, which can provide user federation, strong authentication, user management, and fine-grained authorization.

**Figure 5:**
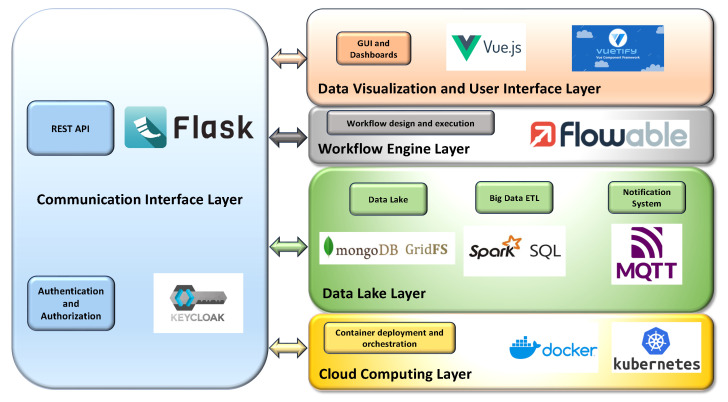
Tools and technologies adopted for the implementation of each functionality provided by the layers of the proposed architecture.

The following Figure 5 summarizes the technologies, tools, and frameworks adopted in each layer and their interactions.

## 6. Use Case

**Figure 6:**
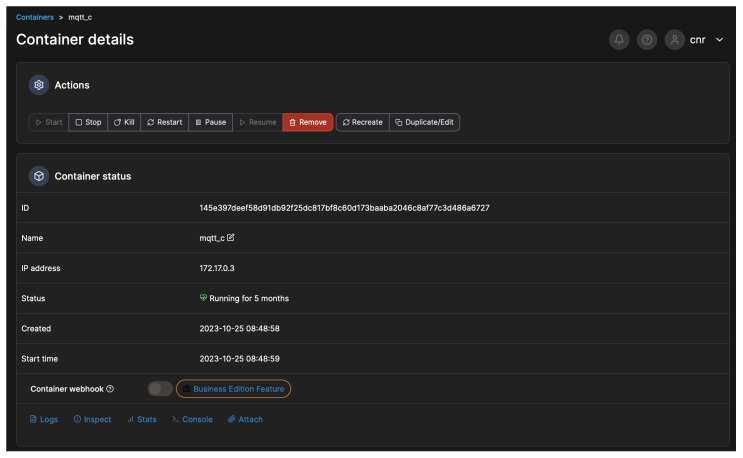
Example of a Docker image managed from the Portainer sandbox.

The IT platform described in this work was designed and developed to support a series of services running in the urban area of Matera, a city in Italy with a high tourist appeal. These services are able to provide analysis, planning, and forecasting functionalities (i.e., environmental parameters, vehicular and pedestrian traffic, 3D interactive city reconstruction, occupancy of the points of interest, energy consumption of buildings, public lighting), as well as implementing applications for the citizens and the tourists (i.e., smart path suggestions, citizen feedback based on interactive geo-localized questionnaires, information on city services and events), thanks to the integration of DTs, AI and ML modules, IoT and CPS devices, a data platform, and the other required subsystems into a single framework. The IT architecture in the use case of Matera was realised on an on-premises cloud infrastructure hosted in a data center in the city of Matera. The Smart Cities setup relied on a container orchestration environment based on Portainer [71], a container management software used to deploy several services at the cloud computing level in the form of microservices and, where this was not possible, as a more complex containerized environment, which leveraged Kubernetes and Docker technologies, as shown in Figure 6, where the container details of the notification module in the Portainer sandbox are depicted.

The UI system deployed and integrated exploiting the proposed architecture acquires and integrates the data produced by the IoT sensor networks installed in the city, the data obtained and/or used from/by the DTs and the other modules (i.e, the city graph representation, city 3D models, geo-localized informative layers, the results of the predictive modules, etc.), and, finally, the data available in public repositories (such as satellite or geographic information), exploiting the resources implemented in the data lake and communication interface layers. The various subsystems installed and deployed in the lower layer of the architecture communicate with each other and share the data using the data lake layer as a data collector. The data integration and ETL functionalities leverage the metadata and the information available in the data catalog, to easily integrate heterogeneous data, extract the information of interest, and transform the data into the specific format suitable for each subsystem.

**Figure 7:**
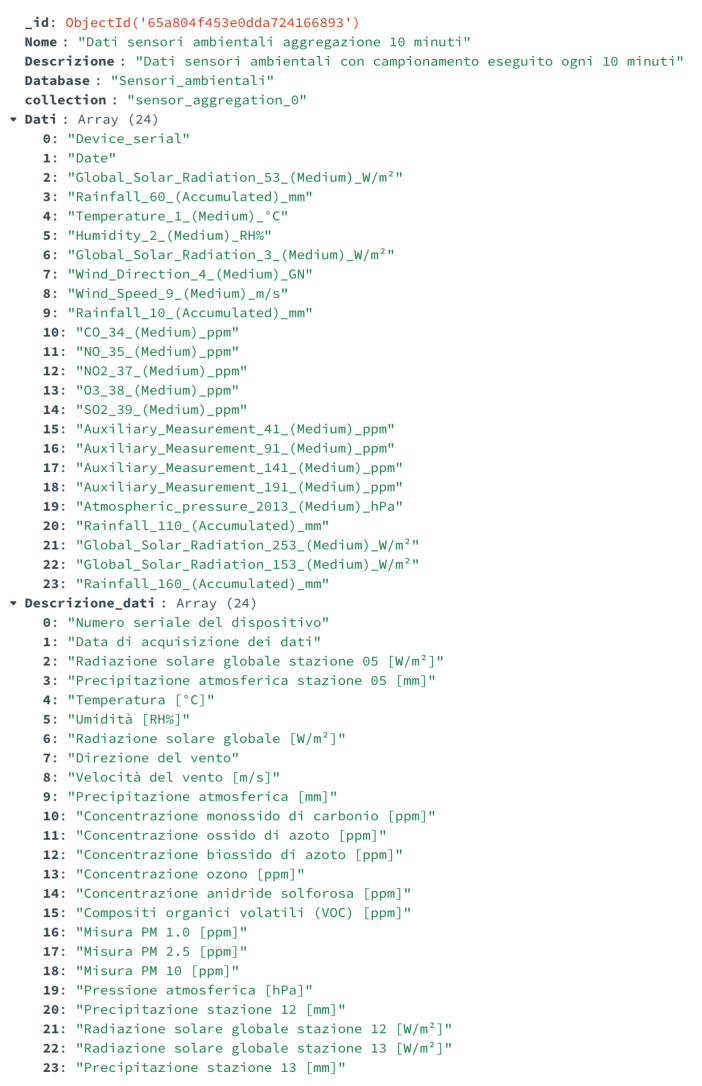
Example of the data catalog: environmental sensor data.

**Figure 8:**
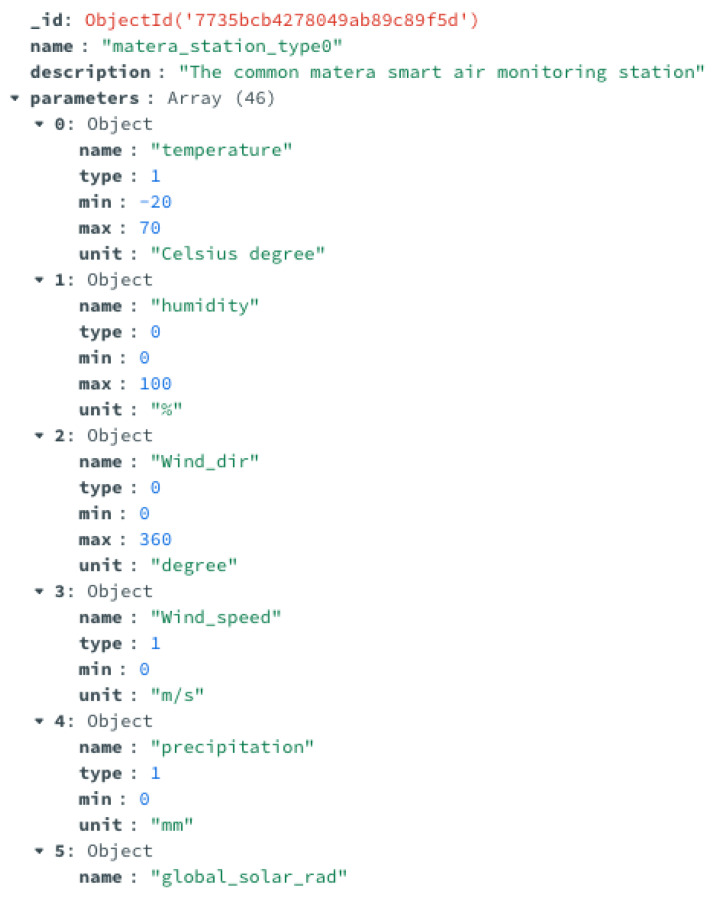
Example of the metadata used for the environmental sensor data.

An example of the ETL of data implemented in the use case is reported in the following Figure 7 and Figure 8. In detail, Figure 7 shows a document of the data catalog related to the data from environmental sensors installed in the city and acquired in the data lake. These data include various measurements, such as rainfall, temperature, wind speed, and direction, etc. This document of the data catalog provides a brief description of the data, the names of the DB and the collection where it is stored, a list of all fields, and the corresponding description. Figure 8 shows some of the metadata added to these sensor data, which include the type (two different kinds of environmental sensors were installed in the city), the minimum and the maximum (if available) values, and the measurement unit.

**Figure 9:**
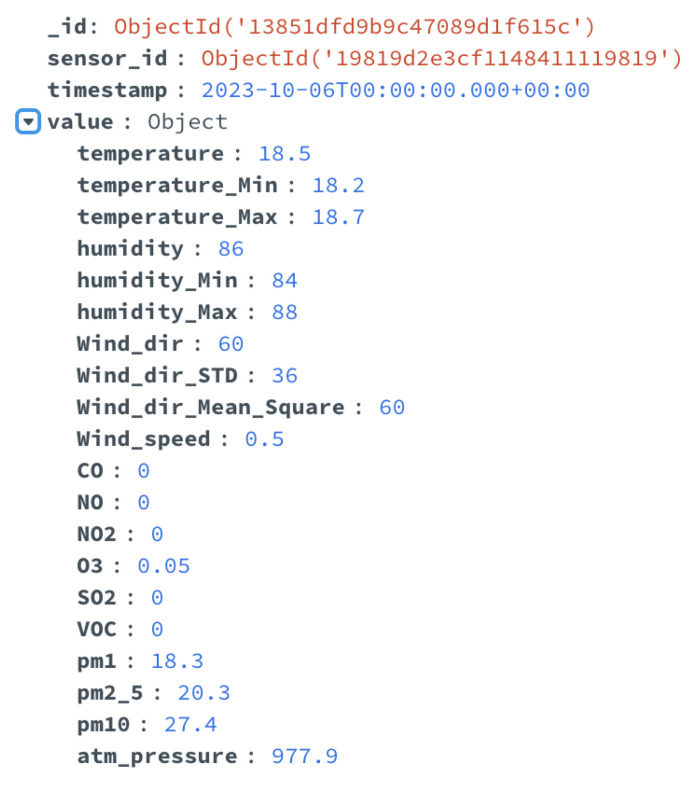
Example of the ETL process applied to environmental sensor data, making the data suitable for the visualization module.

The data integration and ETL functionalities leveraged the metadata and the information available in the data catalog, to easily integrate heterogeneous data, extract the information of interest, and transform the data into the specific format suitable for each subsystem. Figure 9 shows an example of environmental sensor data after the ETL process, applied for use in the dashboard module.

**Figure 10:**
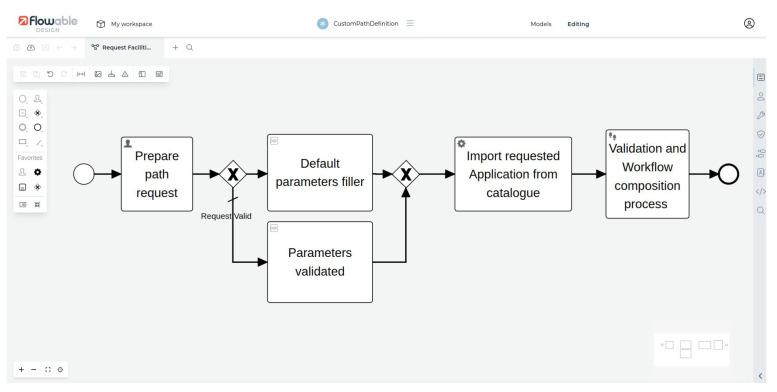
A partial view of a data scientist’s workflow setup during an exploitation of the UI facilities described in Section 4.2.

Authorized users can coordinate and orchestrate these subsystems and the data and process flows using the workflow engine. As an example, Figure 10 depicts the interface of Flowable in the workflow engine layer, with a workflow representing the realization of a data scientist application, in which the initial parameters are collected via a process and, after a validation phase, the workflow as requested by the application users is generated.

**Figure 11:**
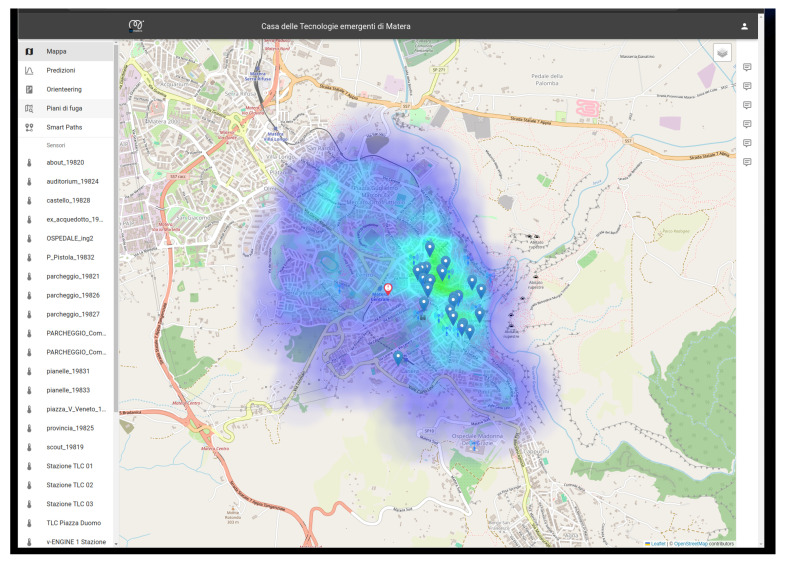
Main window and sensors and POIs view.

**Figure 12:**
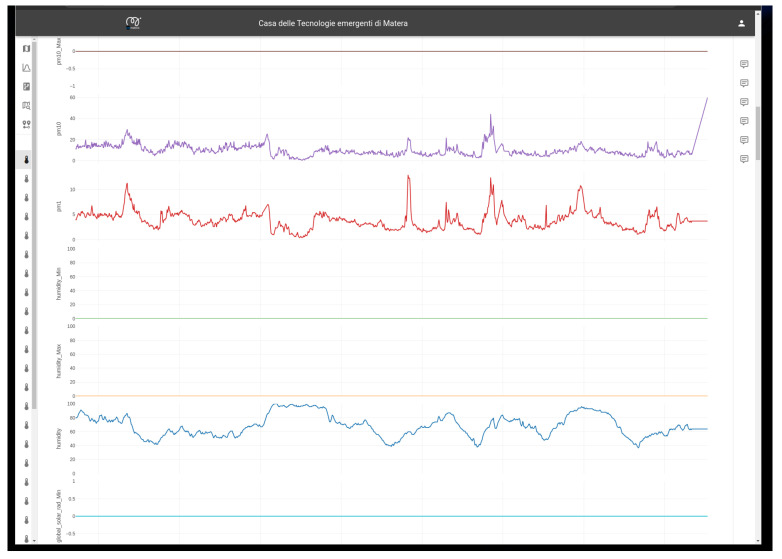
Graphical user interfaces.

Finally, all the static and dynamic information and data available in the data lake are available for the visualization layer and can be easily visualized. Figure 11 and Figure 12 show an example of the implemented GUI and dashboards. Several users have access to the dashboard, seeing different results depending on their individual privileges (e.g., citizens, municipal technicians, etc.), allowing tailored insights and actions. The main window (see Figure 11) serves as a comprehensive dashboard, offering real-time updates on city status, including sensor data, points of interest, and the population distribution across various categories. Population trends are monitored over time, with the heatmap in Figure 11 dynamically updating as the population distribution changes, allowing for informed decision-making and resource allocation. Detailed analyses on specific points of interest provide deeper insights, empowering targeted interventions and optimized city management. The data stored in the Data Lake can be visualized (see Figure 12) and updated in near real time. As mentioned above, the data are also processed by the ETL functionalities included in the data lake layer, exploiting their metadata to automatically extract the required information and provide them to the dashboard in the expected format. In the case of critical events, the system promptly alerts designated personnel, enabling swift response and mitigation measures. Additionally, procedural guidelines generated by the system consider potential interactions among future events, facilitating effective monitoring and optimization of activities tailored to the specific needs of the city. This proactive approach ensures streamlined operations and enhanced resilience in urban management.

The tests performed in this real-world scenario highlighted that the architecture could support all the foreseen functionalities without any issue, as well as being able to fully integrate and coordinate the various subsystems of the UI platform. The requested information was provided respecting the expected performance levels, in terms of data rates and processing times.

## 7. Conclusions

This paper presented the architecture of an IT platform, whose main purpose is facilitating the design, integration, deployment, and management of the subsystems and the data of a UI. To this end, the proposed architecture provides a lower layer that includes the resources for the deployment and orchestration of the container-based implementations of the DTs and other subsystems (analytics, forecasting, etc.) of a complex UI environment. Then, it includes a layer specifically tailored to provide functionalities for the acquisition, storage, sharing, indexing, ETL, and integration of data collected from IoT sensors and data sources external to the UI, as well produced as output by the UI subsystems, acting as a common data collector and facilitating the sharing, integration, and retrieval of data. A workflow engine layer was also included in this architecture, with the purpose of designing and executing workflows for the orchestration and coordination of processes and data flows between the subsystems of the UI required for the analysis and functionalities that involve more modules. Finally, a layer devoted to the visualization of the data and the realization of dashboards was provided. In addition, a transversal communication interface layer provided a standardized communication interface between each layer of the architecture, as well as the UI subsystems and the external system. This interface leveraged a set of REST APIs and also included authorization and authentication mechanisms. Finally, this transversal layer also respects the Italian guidelines for the interoperability of IT systems with public administrations and third parties, following the principles articulated in the New European Interoperability Framework (EIF).

The implementation of the proposed architecture was based on open-source technologies, frameworks, and tools, facilitating its widespread adoption. We tested this architecture in a real-world use case scenario, using it to deploy a UI system in the tourist city of Matera, in Italy. The platform implemented using our architecture allowed us to integrate and run several applications based on DTs and other specific subsystems for the analysis, planning, and forecasting of different aspects of the city, as well as to provide smart services for the citizens and tourists. The tests performed in the use case demonstrated that the architecture was capable of supporting and implementing all the foreseen functionalities, without any issues. Moreover, it allowed the users to collect and integrate all the required heterogeneous data, easily and securely sharing it inside and outside the UI platform. Finally, it also provided the expected performance levels in terms of processing time and data rates.

In summary, this architecture could facilitate the integration and the orchestration of the subsystems of a UI, easing the development and use of a Smart City platform based on open-source technologies and frameworks. It can be easily adapted for the needs of each city by including and integrating additional data and tailored IT modules. Moreover, it also provides functionalities to manage the interactions and process flows among the available subsystems, allowing decision-makers and analysts to create new integrated workflows by exploiting the included workflow engine. These features make it possible to promote the adoption of Smart City platforms, to improve the management of the resources of a city and the well-being of the population.

## Data Availability

The data presented in this study are available on request from the corresponding author.

## Abbreviations

The following abbreviations are used in this manuscript:

IT	Information Technology
UI	Urban Intelligence
DT	Digital Twin
CPS	Cyber-Physical Systems
ETL	Extract, Transform and Load
DB	Database
